# A population-based study on the impact of hospitalization for pneumonia in different age groups

**DOI:** 10.1186/1471-2334-14-485

**Published:** 2014-09-05

**Authors:** Vincenzo Baldo, Silvia Cocchio, Tatjana Baldovin, Alessandra Buja, Patrizia Furlan, Chiara Bertoncello, Francesca Russo, Mario Saia

**Affiliations:** Department of Molecular Medicine, Public Health Section, University of Padua, Istituto di Igiene, Via Loredan 18, 35130 Padova, Italy; EuroHealth Net, Venice, Veneto Region Health Directorate, Dorsoduro 3493, 30123 Venezia, Italy

**Keywords:** Hospitalization, Pneumonia, Elderly, Vaccination

## Abstract

**Background:**

Pneumonia is an important cause of illness and death, particularly in elderly adults. This retrospective study was conducted to estimate the trend of hospitalization for pneumonia in the Veneto from the records of all hospitals in the region (serving a population of 4.81 million) during the years 2004 through 2012.

**Methods:**

The cases of pneumonia identified in the hospital discharge records were all cases in which the first-listed diagnosis was pneumonia, or meningitis, septicemia or empyema associated with pneumonia. The annual total and age-specific hospitalization rates and trends were calculated and correlated with vaccine coverage. Total related costs were also calculated.

**Results:**

There were 110,927 hospitalizations for pneumonia, meaning an annual rate of 256.3/100,000 population, with peaks in children and elderly people. The overall pneumonia-related hospitalization rate did not change significantly during the study period (AAPC: 1.3% [95% CI: −0.5, 3.1]). The rate dropped significantly among the 0- to 4-year-olds, however, from 617.3/100,000 in 2004 to 451.8/100,000 in 2012 (AAPC: −2.5% [95% CI: −4.5; −0.5]), while it increased slightly in adults aged 80+ (AAPC: 1.2% [95% CI: −0.9; 3.4]). The overall pneumonia-related mortality rate was 10.7%. The estimated cost per hospitalized patient was €3,090.

**Conclusion:**

This study shows that hospitalization for pneumonia has a considerable impact on the health services, especially for children and the elderly. No decline in hospitalization rates was seen for the very elderly after the introduction of pneumococcal conjugate vaccination for children.

**Electronic supplementary material:**

The online version of this article (doi:10.1186/1471-2334-14-485) contains supplementary material, which is available to authorized users.

## Background

Pneumonia is an important cause of illness and death, particularly for the elderly. There have been reports of rising numbers of hospital admissions for pneumonia in some industrialized countries [1–4], which may be due to their aging populations and a higher prevalence of concomitant clinical conditions (e.g. chronic obstructive pulmonary disease or diabetes). This trend has recently been reversed, however, since the introduction of pneumococcal conjugate vaccine [5].

The World Health Organization estimates that the most common bacterial cause of pneumonia in adults living in industrialized countries is *Streptococcus pneumoniae* (SP), and pneumococcal infections are considered a major public health issue worldwide for all age groups. The pathogen reportedly identified as being responsible for pneumonia may vary, however, depending on the capacity of the in-hospital laboratory involved, the study design used in reports, the season, and the region where studies are conducted [6].

Pneumococcal pneumonia coincides with bacteremia in 10-30% of patients, in which case it is classified as invasive. It can cause a number of diseases of variable severity, and when the pathogen invades normally sterile sites like the bloodstream and meninges, the resulting forms of pneumococcal disease are again classified as invasive [7]. The other two forms of invasive disease are septicemia and meningitis. Septicemia is a severe bloodstream infection that can rapidly become life-threatening. Patients are generally treated at a hospital’s intensive care unit. The condition is often fatal despite treatment, and survivors are likely to have permanent organ damage, cognitive impairment, and physical disability. As for bacterial meningitis, the epidemiological features of this condition have changed dramatically in recent decades, and the most common cause of bacterial meningitis among younger and older adults is currently SP [8].

The aim of the present study was to estimate the burden of pneumonia in the Veneto Region by assessing the hospital records of pneumonia-related discharges in relation to changes in pneumonia vaccination strategies in the last ten years.

## Methods

A retrospective study was conducted in the Veneto Region (North-East Italy) using the National Surveillance System hospital database. To estimate the annual hospitalization rates for pneumococcal disease, we analyzed the hospital records dating from 1 January 2004 to 31 December 2012 concerning all discharges from all public and accredited private hospitals. In general, the first-listed diagnosis concerned the main condition identified during the patient’s hospital stay, while other diagnoses indicated associated or contributing conditions (comorbidities and/or complications).

During the 9 years considered, the Veneto had an average population of 4.81 million year by year, and an average 0.81 million hospitalizations a year, which decreased from 0.88 to 0.71 million from 2004 to 2012. The mean number of hospital beds was 19,804 with a continuous downward trend from 20,338 in 2004 to 18,892 in 2012 (3.81 hospital beds per 1,000 population). In 2012, the overall hospitalization rate was 136.4 per 1,000 population, for a hospital bed occupation rate of 75% and a mean hospital stay of 7.2 days.

All hospital discharge records containing one of the following codes according to the International Classification of Diseases, Ninth Revision, Clinical Modification (ICD-9-CM) were selected: pneumonia 480.xx-486.xx, 487.0; meningitis 321.xx, 013.0.x, 003.21, 036.0, 036.1, 047, 047.0, 047.1, 047.8, 047.9, 049.1, 053.0, 054.72, 072.1, 091.81, 094.2, 098.82, 100.81, 112.83, 114.2, 115.01, 115.11, 115.91, 130.0, 320, 320.0, 320.1, 320.2, 320.3, 320.7, 320.81, 320.82, 320.89, 320.8, 320.9, 322, 322.0, 322.9; septicemia 038.1x, 038.4x, 003.1, 020.2, 022.3, 031.2, 036.2, 038, 038.0, 038.2, 038.3, 038.8, 038.9, 054.5, 790.7; empyema 510.xx. All pneumonia-related hospitalizations were identified from the hospital discharge records with a first-listed diagnosis of pneumonia or a first-listed diagnosis of meningitis, septicemia or empyema associated with pneumonia in another diagnostic field. We also identified the hospital discharge records using the codes 481.xx, 038.2 and 320.1 in order to further analyze SP-related hospitalization.

Annual total and age-specific hospitalization rates were obtained by dividing the annual number of hospitalizations for pneumonia by the annual population according to the Veneto Regional Authority’s statistical office, expressing the rates as hospitalizations per 100,000 population. For patients readmitted within 30 days only the first hospital stay was considered when calculating the hospitalization rates. The mean days of hospital stay and case-mortality rates (%) were also calculated.

In the Veneto Region, an immunization program with 7-valent pneumococcal conjugate vaccine (PCV7) was first introduced in 2001 as an optional vaccination only for newborn at risk (2–3 and 14 months old), then in 2005 the program was extended to offer vaccination to all children. In 2010, the 13-valent pneumococcal conjugate vaccine (PCV13) was adopted instead of the PCV7 vaccine, and a catch-up program was implemented for infants up to 36 months old. The vaccination coverage rate was <10% from 2004 to 2006 (low PCV7 coverage), <85% from 2007 to 2008 (moderate PCV7 coverage), and >90% since 2009 (high PCV7 coverage up until 2010, then high PCV13 coverage in 2011 and 2012). In the cohort of 65-year-old people, the 23-valent pneumococcal polysaccharide vaccine (PPV23) was optionally offered and reached a low coverage (about 30%) throughout the study period.

We checked the other diagnostic codes to identify the following chronic comorbidities: diabetes mellitus (ICD9-CM code: 250), heart diseases (ICD9-CM codes: 410–414; 402.01, 402.91, 404.01, 404.03, 404.11, 404.13, 404.91,404.93, 452, 428), cerebrovascular diseases (ICD9-CM codes: 430–434, 436–438), respiratory diseases (ICD-9-CM codes: 491.1-491.8; 492–496), malignant neoplasms (ICD-9-CM codes: 140–209), renal diseases (ICD-9-CM codes: 580–588, 591) and dementia (ICD-9-CM codes: 290, 331).

Significant trends over the period considered were assessed as average annual percent changes (AAPC), a summary measure of the trend over a given fixed interval [9]. An AAPC of zero coincides with the hypothesis of a trend that is neither increasing nor decreasing. The 95% confidence interval (95% CI) was calculated and a p value <0.05 was considered significant.

The hospitalized patients’ diagnosis-related groups (DRG), including readmissions within 30 days, were used to estimate the costs of their hospitalization to the healthcare system. The DRGs were calculated with the Core Grouping System Software considering ICD code, age, sex, and resource consumption [10]. Each group has a similar weight in terms of hospital costs and it can be applied to each patient. The total cost was calculated taking the total number of hospital discharges into account. These data may be useful for developing cost-effectiveness models with a view to establishing priorities and strategies, and to assessing the impact of pneumococcal vaccination.

The data obtained from the Regional Statistics Office were analyzed using EPI-Info 2000 software (Center for Disease Control and Prevention of Atlanta, GA, USA) and the Joinpoint Regression Program, rel. 4.0.4. of May 2013 (Statistical Research and Applications Branch, National Cancer Institute, USA).

The study was conducted on data routinely collected by the health services and linked to anonymized records that make it impossible to identify the individuals concerned. Data in the Local Health Authority registries are recorded with the patient’s consent and can be used as aggregated data for scientific studies without further authorization [11]. This study complies with the Helsinki Declaration and with Italian privacy law n. 196/2003 on the protection of personal data and it was approved by the Ethical Committee of Padua Hospital.

## Results

Overall, we identified 116,198 hospitalizations associated with pneumonia. After omitting the 5,271 (4.5%) readmissions within 30 days, the remaining 110,927 included 108,278 (97.6%) for which the first diagnosis was pneumonia. In the other cases associated with pneumonia, the first diagnosis was septicemia in 2,014 (1.8%), meningitis in 388 (0.3%) and empyema in 247 (0.2%).

**Figure 1:**
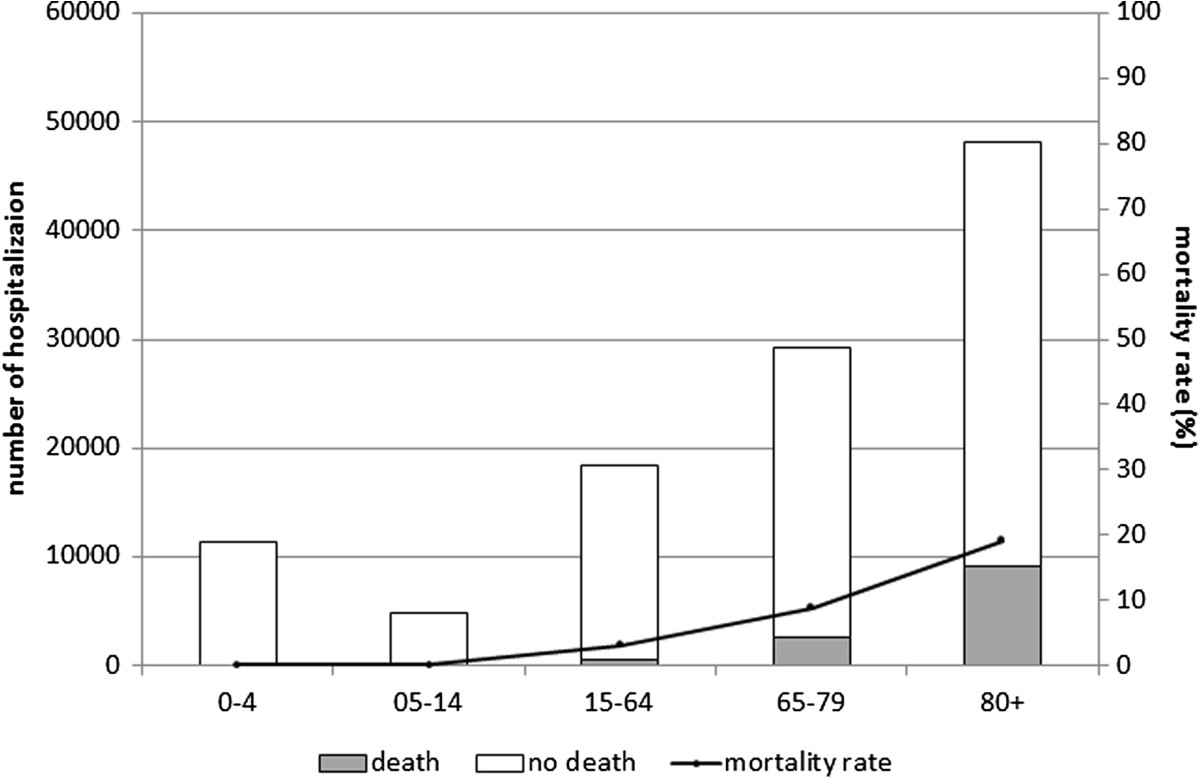
**Number of hospitalizations and mortality rate by age group.**

**Figure 2:**
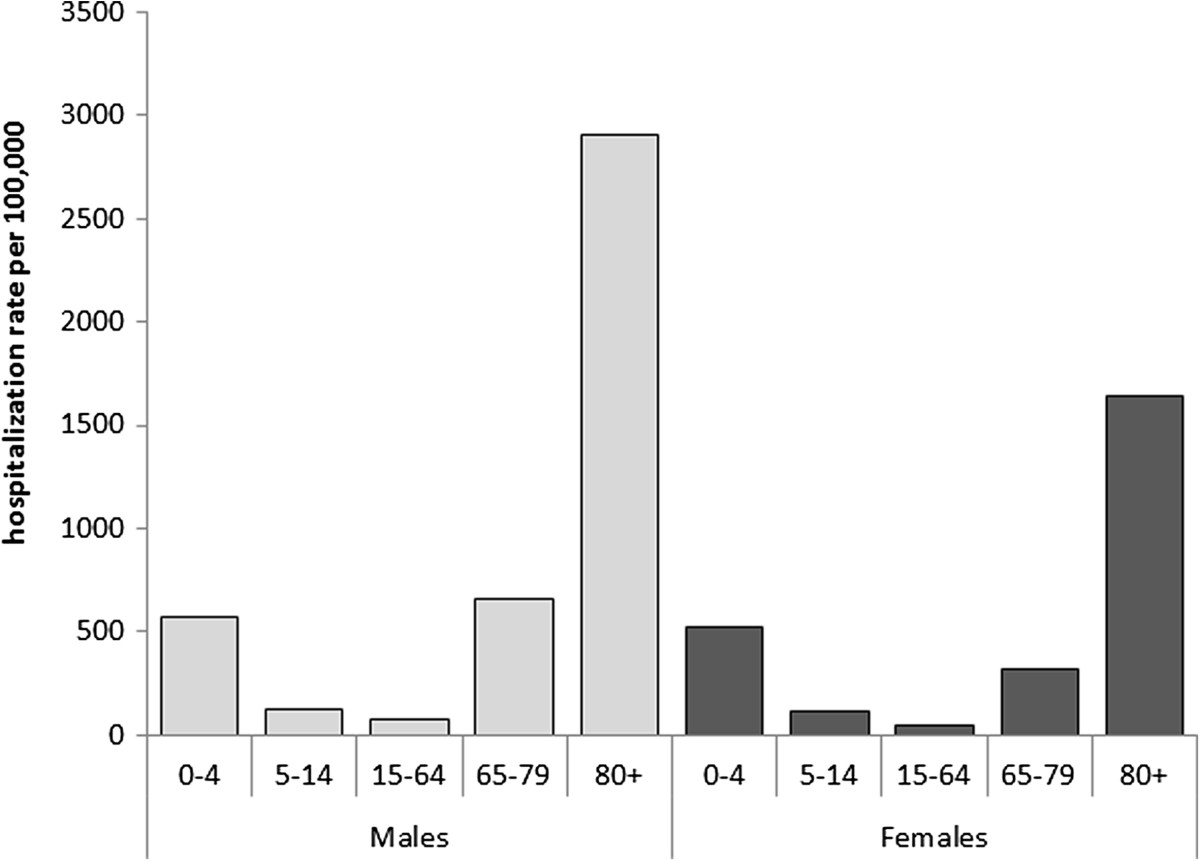
**Pneumonia-related hospitalization rate in the Veneto Region by age group and gender (2004–2012).**

**Figure 3:**
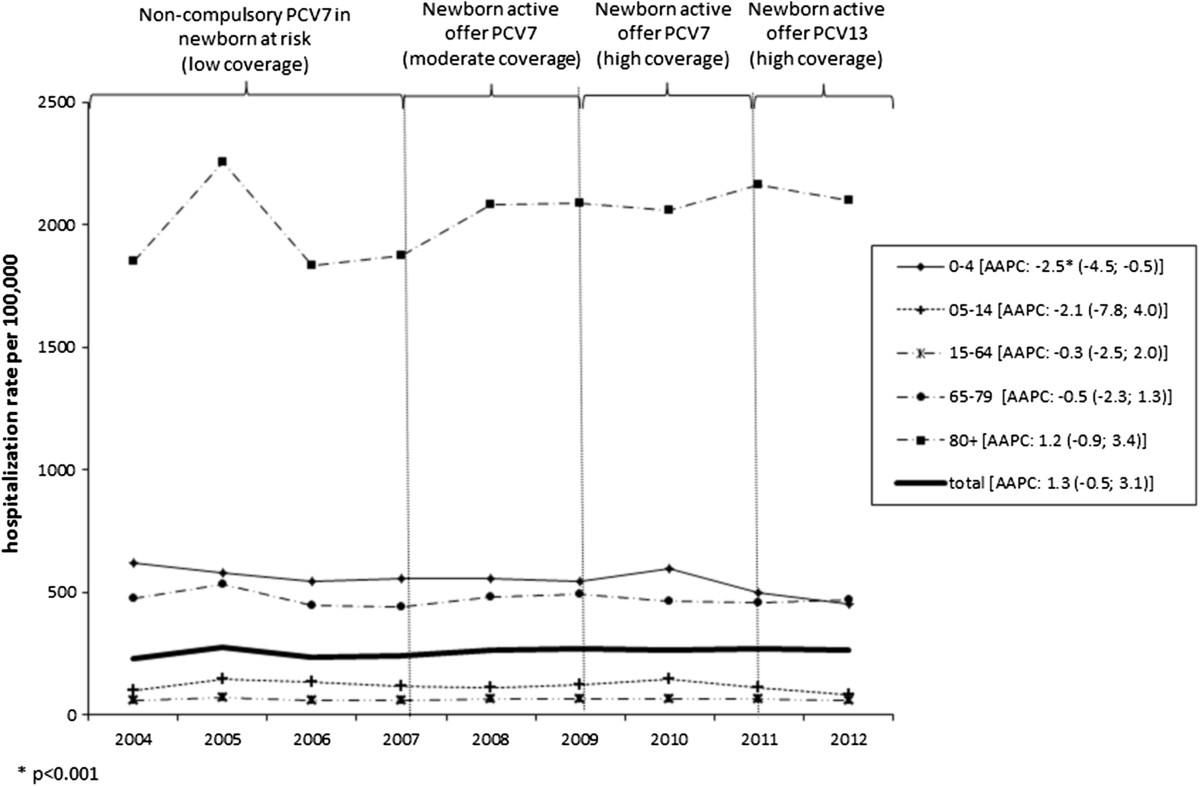
**Pneumonia-related hospitalization rate (per 100,000 population) in the Veneto Region by age group (2004–2012).**

The characteristics of the sample are shown in Table 1. Overall, 53.6% of the patients were male (59,442 cases) and the highest proportion of hospitalizations was recorded among ≥65-year-olds (68.9%). Patients were Italian in 95.6% of cases. In the sample as a whole, 46.4% of patients had at least one chronic comorbidity, and comorbidities tended to increase with age. The overall average hospital stay was 11.6 ± 10.1 days, and did not vary between genders. The average hospital stay increased with age, reaching 13.2 days for patients aged 65 or more. The estimated overall annual cost of these pneumonia-related hospitalizations was approximately €40 million, with an estimated cost per patient of €3,090. People over 65 accounted for 67.6% of the estimated overall pneumonia-related cost (€27 million in all, corresponding to €28.7 per person over 65 in the general population). As for outcome, the overall pneumonia-related mortality rate was 10.7% and remained stable during the study period. This rate increased with age, peaking in people over 80 (18.6%) (Figure 1). The annual pneumonia-related hospitalization rate during the study period was 256.3 per 100,000 population (280.8/100,000 males and 232.9/100,000 females). Both genders showed two age-related peaks, one in children aged 0–4 years, the other in the elderly, with four-fold higher rates among people aged 80 or more (Figure 2).The overall pneumonia-related hospitalization rate increased slightly, but not significantly between 2004 and 2012, from 232.0/100,000 in 2004 to 262.2/100,000 in 2012 (AAPC 1.3% [95% CI: −0.5, 3.1]). The rate remained fairly constant in the age groups from 5 to 79 years old throughout the study period (p = n.s.), but it dropped significantly among the 0- to 4-year-olds, from 617.3/100,000 in 2004 to 451.8/100,000 in 2012 (AAPC: −2.5%; [95% CI: −4.5; −0.5]), while it rose though not significantly, for adults aged 80 or more (AAPC: 1.2%; [95% CI: −0.9; 3.4]) (Figure 3).

**Table 1:** **Hospital admissions for pneumonia in the Veneto Region by patients’ characteristics and age group (2004–2012)**

	Total		Age groups									
Characteristics			0-4		5-14		15-64		65-79		80+	
	(n. 110,927)		(n. 11,430)		(n. 4,765)		(n. 18,299)		(n. 28,886)		(n. 47,547)	
**Gender [n (%])**												
Males	59,442	(53.6)	6,151	(53.8)	2,575	(54.0)	11,266	(61.6)	18,181	(62.9)	21,269	(44.7)
Females	51,485	(46.4)	5,279	(46.2)	2,190	(46.0)	7,033	(38.4)	10,705	(37.1)	26,278	(55.3)
**Nationality [n (%])**												
Italian	106,031	(95.6)	9,382	(82.1)	4,147	(87.0)	16,546	(90.4)	28,583	(99.0)	47,373	(99.6)
Foreign	4,896	(4.4)	2,048	(17.9)	618	(13.0)	1,753	(9.6)	303	(1.0)	174	(0.4)
**First listed diagnosis [n (%])**												
Pneumonia	108,278	(97.6)	11,270	(98.6)	4,704	(98.7)	17,587	(96.1)	28,003	(96.9)	46,714	(98.2)
Septicemia	2,014	(1.8)	79	(0.7)	20	(0.4)	424	(2.3)	714	(2.5)	777	(1.6)
Meningitis	388	(0.3)	36	(0.3)	11	(0.2)	183	(1.0)	130	(0.5)	28	(0.1)
Empyema	247	(0.2)	45	(0.4)	30	(0.6)	105	(0.6)	39	(0.1)	28	(0.1)
**Comorbidity [n (%])**												
Heart diseases	19,163	(17.3)	3	(0.0)	3	(0.1)	695	(3.8)	5,229	(18.1)	13,233	(27.8)
COPD and asthma	11,466	(10.3)	366	(3.2)	141	(3.0)	1,232	(6.7)	4,321	(15.0)	5,406	(11.4)
Diabetes mellitus	11,302	(10.2)	3	(0.0)	10	(0.2)	1,478	(8.1)	4,443	(15.4)	5,368	(11.3)
Dementia	9,393	(8.5)	0	(0.0)	0	(0.0)	119	(0.7)	1,933	(6.7)	7,341	(15.4)
Stroke	7,511	(6.8)	2	(0.0)	2	(0.0)	257	(1.4)	2,023	(7.0)	5,227	(11.0)
Renal disease	6,892	(6.2)	19	(0.2)	28	(0.6)	622	(3.4)	2,030	(7.0)	4,193	(8.8)
Cancer	5,951	(5.4)	27	(0.2)	30	(0.6)	975	(5.3)	2,625	(9.1)	2,294	(4.8)
**Hospital stay (days [SD])**	11.6	(10.1)	4.7	(3.6)	5.1	(3.7)	10.9	(10.0)	13.2	(10.1)	13.2	(10.6)
**Hospitalization cost/year [million €]**	40.0		4.5		1.9		6.6		10.5		16.5	

*COPD: chronic obstructive pulmonary disease.*

The diagnosis in the hospital discharge records referred specifically to SP in 2.4%, 87.9%, 21.0% and 13.8% of cases of pneumonia, meningitis, septicemia and empyema, respectively. Patients were male in 54.9% of cases; the mean age was 51.2 ± 31.7, and the in-hospital mortality rate was 8.3%.

The age-specific pneumonia-related hospitalization trend was much the same as the overall pneumonia-related hospitalization trend: for infants aged 0–4 years, it was stable from 2004 to 2008, then dropped from 49.9/100,000 in 2009 to 23.3/100,000 in 2012 (p = n.s.); the trend remained stable in the age groups from 5 to 79 years (hospitalization rate 5.5/100,000); and it rose for adults aged 80 or more, from 18.6/100,000 in 2004 to 40.1/100,000 in 2012 (p = ns).As concerns PCV coverage and the type of vaccine used, the pneumonia-related hospitalization rates were highest for the youngest and oldest age group throughout the study period, the rates for 0- to 4-year-olds being similar to those among 65- to 79-year-olds (around 500/100,000). In 2011–2012, the hospitalization rates dropped for infants aged 0–4 and children aged 5–14, while they remained stable for adults aged 65 or more (Figure 3).

## Discussion

It is not easy to estimate the morbidity of pneumonia in the general population. This is partly because the currently-available epidemiological picture is hazy due to differences in study designs, population profiles, access to healthcare, and registration methods. There is also the fact that most patients developing pneumonia in our Region are not admitted to hospital, so the pneumonia-related hospitalization rates are bound to underestimate the burden of the disease. The hospital records can nonetheless provide a picture of the more severe cases [12].

The present study examined pneumonia-related hospital admissions in a large cohort and the results show that this disease places a heavy burden on hospital resources and is an important public health issue. The overall pneumonia-related hospitalization rate of 256.3/100,000 identified during the study period showed a slight predilection for male gender (with 1.2 times as many cases as among females), and confirmed that children and the elderly are at higher risk of hospitalization for pneumonia than younger adults. Among patients over 85, pneumonia is also associated with co-morbidities and a mortality rate that make it difficult to manage outside the hospital.

The Veneto Region has achieved a greater accuracy in diagnosing pneumonia by revising its guidelines on the ICD-9-CM codes used in hospital discharge records in 2010: this may have led to an underestimation of the drop in the number of cases among children and an overestimation of the rising figures for the elderly over the last two years of our study period [10]. Nonetheless, our data revealed a significant decline in the hospitalization rate for pneumonia among children in recent years, coinciding with the introduction of PCV13 in lieu of PCV7, which provides coverage for six more serotypes, including 3, 1, 19A and 7 F, and its adoption was also associated with a catch-up strategy [13]. A significant reduction in hospitalization rates for pneumonia generally, and for pneumococcal pneumonia was reported for the first time in Italy among children aged 0–24 months in Liguria [14], and also more recently in the Apulia Region [15].

In 20-60% of cases, community-acquired pneumonia is attributable to SP, although only a small percentage of these cases are identified as such [16] (it is estimated that, when the microorganism is not specified, about 36% of cases are attributable to SP infection [6]). In our sample, SP was specifically diagnosed in 2.4% of cases; this proportion varied depending on the first-listed diagnosis, being lower for pneumonia and higher for meningitis. A recent review on the burden of community-acquired pneumonia in Europe found that Italy had the lowest pneumococcus identification rate. This points to the need to improve the tests used to diagnose pneumonia because a more accurate pneumococcus detection would provide a clearer picture of the true burden of community-acquired pneumonia [6].

As reported by other Authors [5], we found no declining hospitalization rate for the most elderly (80+), and this epidemiological picture could be due to various factors. The magnitude of the indirect effects of vaccine-derived immunity depends on multiple influences relating to the transmissibility of the infection, the nature of the vaccine-induced immunity, the patterns of mixing and transmitting infections in a given population, the distribution of the vaccine and, more importantly, the population’s immunity. The nuances of immunity and the complexity of population heterogeneity make predictions difficult [17, 18]. The Veneto Region’s active surveillance system for monitoring invasive bacterial diseases shows that serotypes not covered by the 7-valent pneumococcal conjugate vaccine, such as 19A, play a significant part [19], suggesting the likelihood of a reversal of the herd effect [20].

The risk of pneumonia increases considerably with age and it is important to ensure a good vaccination coverage for both influenza and pneumococcus among older people. In the latter period of our study, the annual influenza vaccination coverage of the elderly population dropped from about 75% in 2004 to about 60% in 2012, meaning that other measures are needed to prevent pneumonia in seniors [21]. Immunization with PPV23 has been implemented differently in our area in recent years, generally reaching very low overall coverage rates even among people with medical indications. In addition, trials conducted on PPVs have failed to provide evidence to support the routine use of PPV to prevent all-cause pneumonia or mortality, and some studies indicate that PPVs confer little protection against invasive pneumococcal diseases [22–24]. Prevention remains the most valuable tool to help reduce the burden of pneumonia, and vaccination strategies can be used for the primary prevention of infection and possibly to reduce this burden in all age groups. The availability of new-generation pneumococcal conjugate vaccines with a broader antigenic spectrum and suitable for people of all ages suggests interesting new opportunities for improving the control of pneumococcal disease in the population as a whole. This strategy has had a significant impact on the burden of pneumonia in areas where the vaccines have been introduced and a good coverage has been achieved (5, 14–15). The introduction of PCV13 for our Region’s newborn in 2010, and its subsequent use in people over 65 years old [13, 25] can be expected to produce some benefits in years to come. A better understanding of the effects of higher-valence vaccines can improve the sensitivity of surveillance systems and add to our knowledge of the potential invasiveness of non-PCV serotypes, as measured by comparing the prevalence of their carriage with their prevalence in invasive pneumococcal disease. This is an aspect of surveillance that has hitherto been largely overlooked.

A drawback of using hospital discharge records lies in the difficulty of distinguishing between community-acquired pneumonia and hospital-acquired pneumonia. Our data (for more than 97% of our sample) are based on first admissions for pneumonia, and most cases were probably community-acquired because hospital-acquired infections are not normally recorded as a first diagnosis. We only considered admissions for which pneumonia was a secondary diagnosis (2.4% of the sample) if the first diagnosis was septicemia, meningitis, or empyema. This was done to assess the potential impact of vaccination, which might be more effective on community-acquired pneumonia and less so on hospital-acquired pneumonia, because of the different types of agent pathogen involved [26, 27].

## Conclusions

In conclusion, this study shows that hospitalization for pneumonia has a considerable impact on the health services, especially for children and the elderly. We identified a reduction in the pneumonia-related hospitalization rate in children, especially in the latter part of the period considered, while it increased slightly in the elderly. The most appropriate prevention measures need to be defined to deal with these epidemiological patterns of hospital admissions for pneumonia to prevent worsening outcomes and higher costs in future.

## Authors’ original submitted files for images

Below are the links to the authors’ original submitted files for images. Authors’ original file for figure 1 Authors’ original file for figure 2 Authors’ original file for figure 3
