# Supplementary figures and images for: A population-based study on the impact of hospitalization for pneumonia in different age groups
Source: BMC Infect Dis. 2014 Sep 5;14:485. doi: 10.1186/1471-2334-14-485 (PMC4164793; doi:10.1186/1471-2334-14-485)

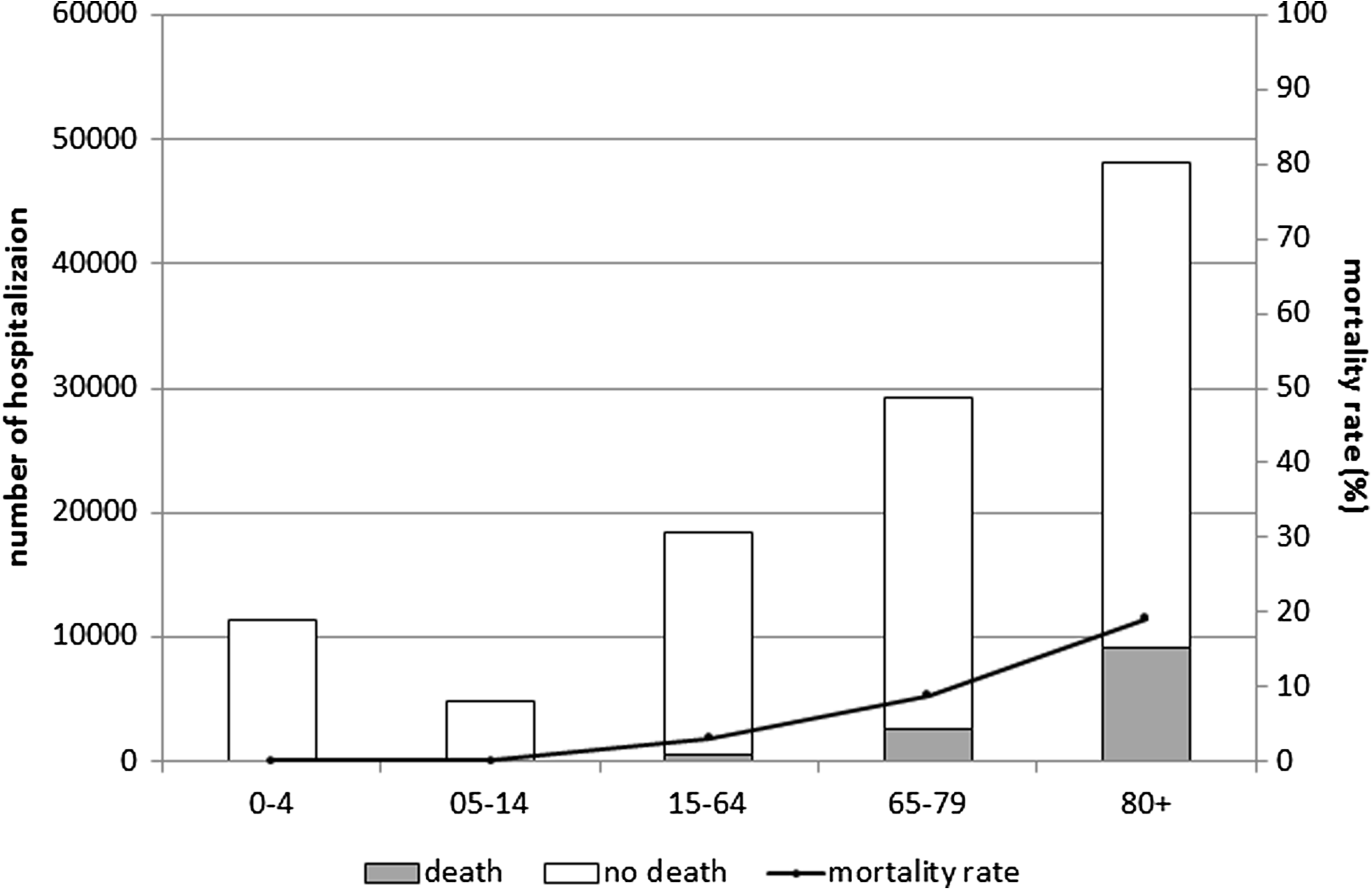

Supplement: Supplementary file 1 — Authors’ original file for figure 1 [file 12879_2014_3790_MOESM1_ESM.tif]

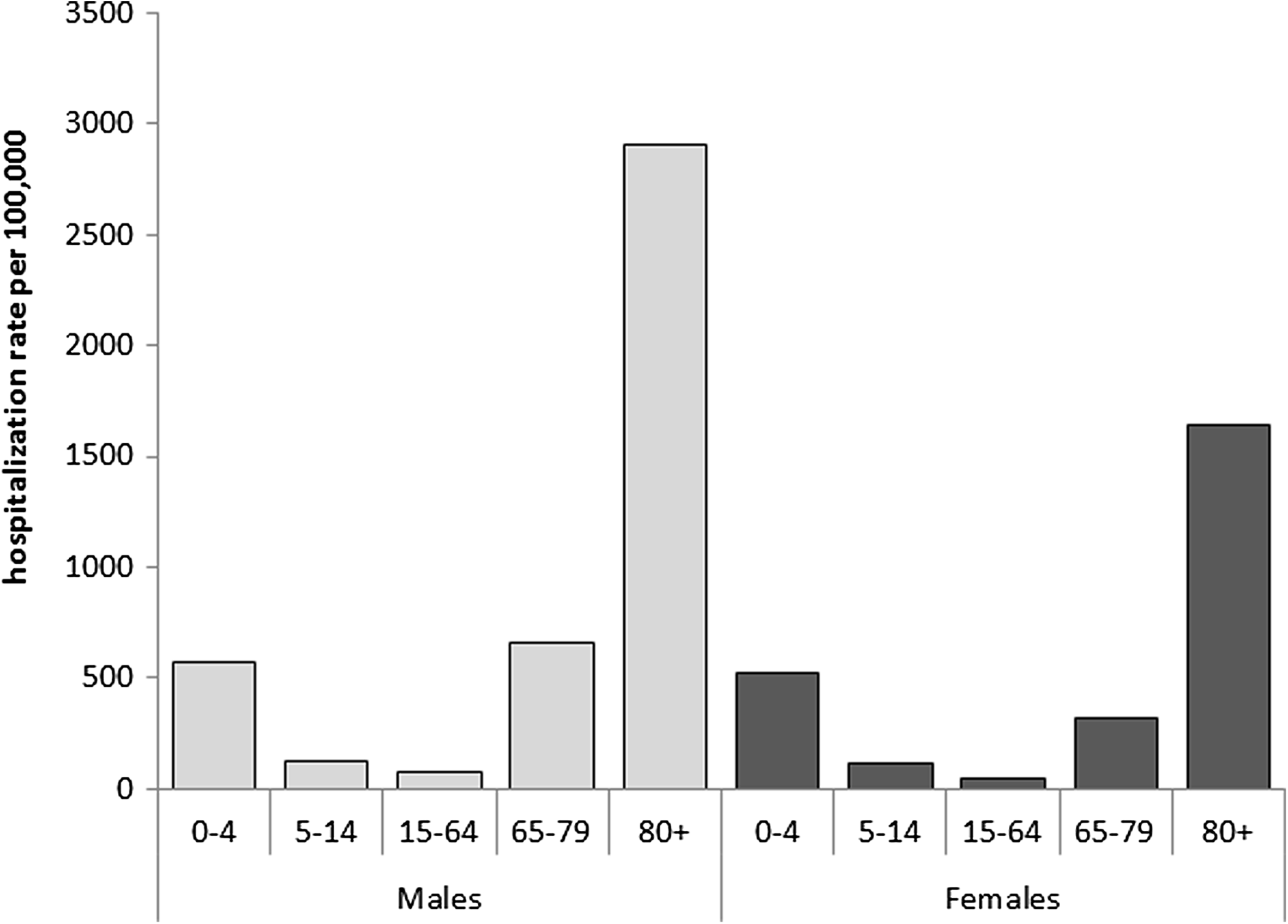

Supplement: Supplementary file 2 — Authors’ original file for figure 2 [file 12879_2014_3790_MOESM2_ESM.tif]

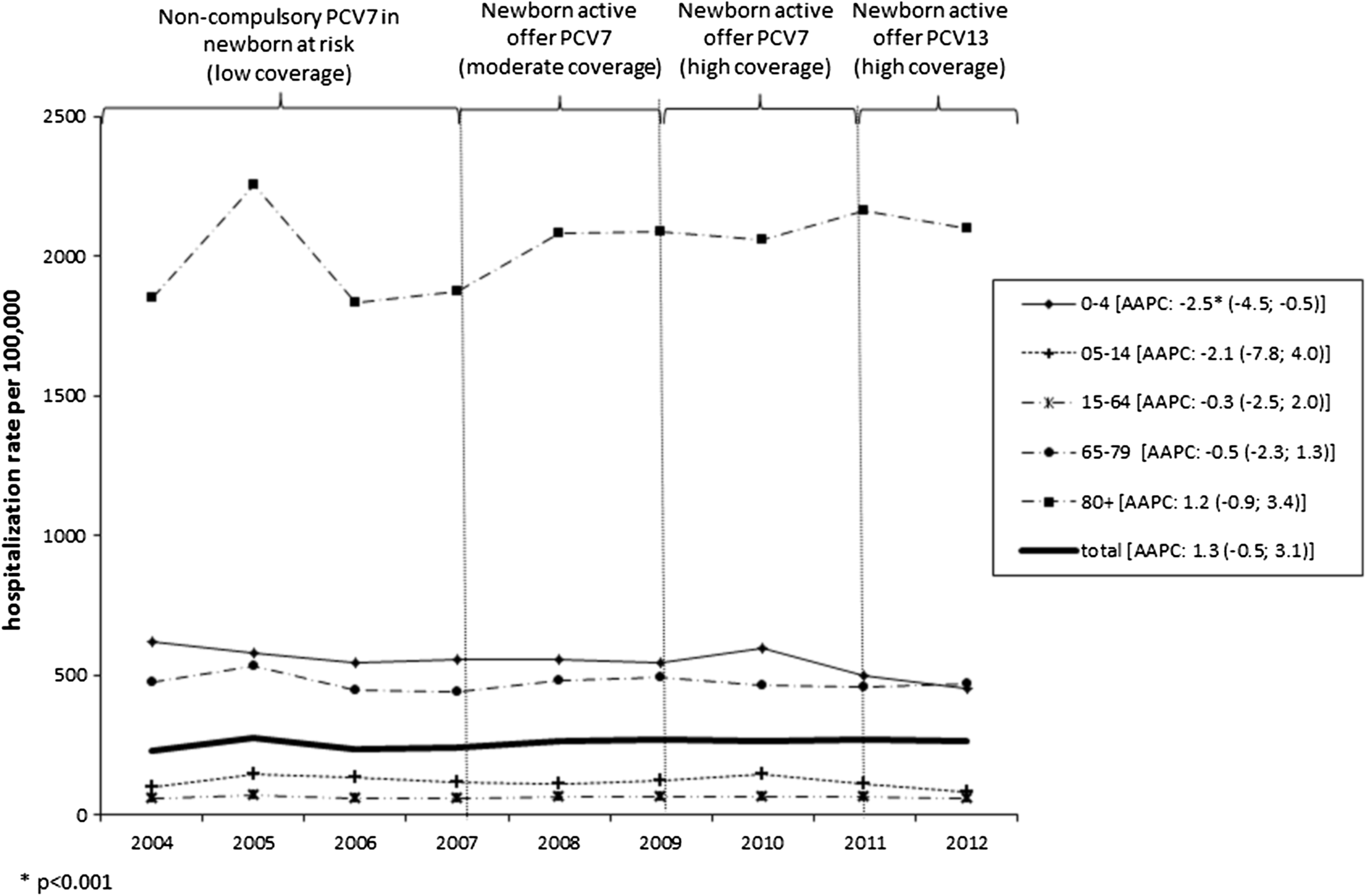

Supplement: Supplementary file 3 — Authors’ original file for figure 3 [file 12879_2014_3790_MOESM3_ESM.tif]
